# Touching Matters: Affective Entanglements in Coronatime

**DOI:** 10.1177/1077800420960167

**Published:** 2021-09

**Authors:** Vivienne Bozalek, Denise Newfield, Nike Romano, Lieve Carette, Katharine Naidu, Veronica Mitchell, Alex Noble

**Affiliations:** 1 University of the Western Cape, Cape Town, South Africa; 2 Rhodes University, Grahamstown, South Africa; 3 University of the Witwatersrand, Johannesburg, South Africa; 4 Cape Peninsula University of Technology, Cape Town, South Africa; 5 Ghent University, Belgium; 6 University of South Africa, Pretoria, South Africa; 7 University of Cape Town, South Africa

**Keywords:** touch, matters, affect, reading group, virtual, the void, potential, coronatime, entanglements

## Abstract

This article troubles touch as requiring embodied proximity, through an affective account of virtual touch during coronatime. Interested in doing academia differently, we started an online Barad readingwriting group from different locations. The coronatime void was not a vacuum, but a plenitude of possibilities for intimacy, pedagogy, learning, creativity, and adventure. Although physically apart, we met daily through Zoom, and we touched and were touched by each other and the texts we read. A montage of writing fragments and a collective artwork, based on the Massive_Micro project, highlight virtual touching. Undone, redone, and reconfigured, we became a diffractive human/nonhuman multiplicity.

The world seemingly went silent, schools and universities closed, we were told to stay home in an unprecedented global pandemic and subsequent lockdown. COVID-19 took over, shifting established habits and practices and catalyzing new ways of being, doing, and thinking. At the southern tip of Africa, we, a group of seven academics, created an online space to connect with one another in generative ways across Cape Town, Johannesburg, and Brugge. During these troubling, isolating coronatimes, the online sessions provided opportunities for us to touch and be touched by others, both human and nonhuman. In what follows, we describe and expand on our thinking around virtual touching as an affective force that permeated our intra-actions.^1^

Our aptly named Corona Reading Group began when the coronavirus made itself known to us in South Africa, and had already made its presence felt in Belgium. As members of the National Research Funded project entitled, *Doing Academia Differently*, we came together from disparate fields including architecture, health sciences, disability studies, art history, English literature, and higher and school education. Through our multiple research endeavors that include PhD studies, we formed an online reading group.

We are reading Karen Barad’s (2007) foundational book, *Meeting the Universe Halfway* (MUH). We feel connected by our common desire to understand Barad’s relational ontology that encourages different ways of thinking and doing research. We grapple with the intricacies of Barad’s account of quantum field theory (QFT) and her concepts of in/determinacy, intra-action, diffraction, becoming-with, the void, and touch. These concepts have the potential to contribute toward transforming pedagogical practice.

Our ongoing online sessions together/apart^2^ diffract^3^ COVID-19 concerns through Barad’s work, creating new sensibilities in troubling times. This inclusive, expansive, and touching process reaches beyond traditional academic interactions around texts that tend to be contained within conventional reading and writing events. Although reading Barad’s work is central to our meetings, the process has also allowed opportunities for reciprocity involving the sharing of images, music, stories, celebrations, and participation in the Massive and Microscopic Sensemaking project (Markham & Harris, 2020). Ocean waves in Cape Town and Belgium diffract through moments of wonder and delight, amid the anxiety and fear of proliferating COVID morbidities, as well as the stark foregrounding of racialized and economic inequalities and suffering.

## Matter and Mattering

Barad is one of the feminist new materialists who points out that during the linguistic turn, *matter* fell from grace, with language taking precedence in the construction and interpretation of reality. Barad (2007) brings back the importance of matter as both ontological and ethical. She proposes that matter matters—it is both of substance and significance. She does not, however, eschew the importance of discourse, but rather sees matter and discourse as inextricably entangled. She proposes that we do not need language to access the material world. This is a performative view of the world, foregrounding doings, practices, and actions, rather than a representationalist one, where the material world is accessed through description. Furthermore, matter is not passive and inert, but a lively participant in the world’s becoming. Matter is described as a doing or a “congealing of agency” (Barad, 2007, p. 151). Barad’s (2007) agential realist understanding of matter is that it is “a dynamic and shifting entanglement of relations, rather than . . . a property of things” (p. 35). Her approach foregrounds matters of ontology, ethics, and epistemology—being, doing, and knowing, matters of fact and matters of concern and care. Science, meanings, and values are all entangled with touch: “[v]alues and facts are cooked together as part of one brew” (Barad in interview with Juelskjær & Schwennesen, 2012, p. 16).

The title of this article, “touching matters,” points to the double meaning of matter discussed above, namely, substance in its ongoing materialization, and what matters from an ethical perspective. Our title uses matter as a noun and as a verb.

## Virtual Touch and Affect

This article performs an affective account of touch during coronatime, a period that flashed up without warning, like past plagues. It focuses on touching in a time of No Touching. Touch is commonly defined in terms of physical contact—as when a body is in contact with another body. The complex association of touch with sin and contamination in many religious traditions and cultural practices has now re-emerged, in contradistinction to its positive associations with warmth and intimacy. In COVID times, where the virus is thought to be spread by aerosol transmission or contaminated surfaces, touching is discouraged, feared, and shunned. South African President Cyril Ramaphosa’s declaration of a National State of Disaster on March 15, 2020, enforced a series of no-touching measures recommended by the World Health Organization—social distancing; the wearing of masks; use of disinfectants; closure of educational, work, and recreational spaces; and, to boot, the banning of sales of alcohol and cigarettes. Our Corona Reading Group has thus been meeting online, on Zoom, for 2 hours a day, 6 days a week, for 5 months.

The notion of touch has been addressed by a range of scholars in the field of digital culture. Although taking different perspectives, they share an interest in touch in online spaces and technological environments. Ahmed and Stacey (2003) draw on Merleau-Ponty’s depiction of embodiment as fleshy, material, and open to other bodies. Marks’s (2002) concept of “haptic visuality” in film and video draws on Deleuze and Guattari’s preference for the term “haptic” over “tactile,” on account of its linking of the optical with touch. Sundén (2014) explores the politics of digital touch in popular culture. Jewitt et al.’s (2020) recent book reports on the InTouch project. Focusing on the nature, design, and potentials of digitally mediated touch, the book argues that digital touch moves beyond ways of seeing to include new ways of feeling. While our article brushes up against this scholarship in several respects, we take a different route. Thinking with Karen Barad, Brian Massumi, and Erin Manning (whose own work follows that of Spinoza, Deleuze and Guattari, and Whitehead and Derrida), we take an alternative pathway to the question of virtual touch in coronatime.

According to Barad (2014) “[i]n an important sense, touch is the primary concern of physics” (p. 153). However, the materiality of touch is not a simple affair. Her agential realist account of the world is founded upon relationality, the basis of all matter—living and nonliving. QFT deconstructs the reductionist essentialism of classical physics, radically undoing identity and determinacy, through its propositions about the touching of physical and virtual particles in the void. Virtuality here refers to the indeterminacy of being and nothingness—virtual particles are both there and not there. As Barad (2019) puts it:

Virtual particles do not traffic in the metaphysics of presence. They do not exist in space and time. They are ghostly non/existences that teeter on the edge of the infinitely fine blade between being and non-being (p. 529).

For Barad, contrary to the classical notion of the void being a lack or nothingness, vacuum fluctuations inside this void give rise to life. Barad (2019) has a vitalist notion of energy, life-generation, and decay. What seems to be a vacuum or void surrounding the electron is actually not empty, but full of lively play and desires. Physical and virtual particles touch each other in an indeterminate and infinite set of possible intra-actions (Barad, 2015, p. 158). For example, electrons can emit photons and then reabsorb them; in this way, they simultaneously absorb the other and touch themselves. Thus, touching the self puts one in touch with alterity, “the stranger within” (Barad, 2019, p. 532).

Touching in the void is hauntological; it occurs in an entangled sense of time, where each moment is in superposition with all other moments—an “infinite multiplicity” (Barad, 2019, p. 525). The noncontemporaneity and virtuality of touch allow us to understand how bodies are both haunted and touched by ghosts from the past and marked by traces (Barad, 2019; Derrida, 1994).

Touch occurs at all scales. Inside the atom, electrons generate and exchange energies with other particles: “Electrons are charged particles, which means that they are susceptible to, or we might even say inclined toward, touching and being touched” (Barad, 2017, p. 79). To evoke nature’s desire for touching, Barad (2015) uses the spectacular phenomenon of lightning. She paints a vivid picture of a stormy sky:

Deep darkness, without a glimmer of light to settle the eye. Out of the blue, tenuous electrical sketches scribbled with liquid light appear/disappear faster than the human eye can detect. Desire builds, as the air crackles with anticipation. Lightning bolts are born of such charged yearnings . . . Lightning is an energizing play of a desiring field (p. 387).

Lightning begins tentatively with electrically charged stepped leaders that gesture toward earth, after which oppositely charged streamers respond upward: a touching between earth and clouds. What is at issue here is the nature of matter and its agential capacities for imaginative, desiring, and affectively charged forms of bodily engagements. Our article explores how, like lightning, our Corona Reading Group is “an exploration of charged yearnings and the sparking of new imaginaries” (Barad, 2015, p. 387) in the thick-now of the present (Benjamin cited in Barad, 2019, p. 26).

Brian Massumi’s (2015) work on affect puts forward the idea of touching as affecting and being affected, what a body can be or do, the capacity to act and be acted upon. Affect involves the transitions a body takes when it steps over a threshold. For Massumi, affect is a dimension of life enacted in processes, such as reading and writing. The virtual for Massumi (2011), as for Deleuze, is synonymous with potential, and more particularly, event potential. It is not another plane of reality, rather it “composes reality and, in itself, contains all possible realities” (Young et al., 2013, p. 330). The virtual can thus open up, as enthused by Guattari, “fabulous possibilities of liberation” (Guattari in Genosko, 2009, p. 87). Our virtual touching as sensory, intimate, and of the body was primarily about intensities, moving toward the other, and being open to the more-than.

Manning (2007), like Barad and Massumi, stresses touch as a bodily thing. She sees the senses as prostheses which expand the body and its abilities, and she conceptualizes touch as a moving toward, a desire for communication and intimacy: I reach out to touch you and you receive my touch; then you reach out to touch me. This reaching across bodily boundaries is risky and uncertain, since the response cannot be determined in advance.

For Manning, touch is a synesthetic sensory encounter, operating in dialogue with the other senses. Touch involves a pact between bodies in metamorphosis as they respond to one another in the context of other stimuli, human, nonhuman, aesthetic and political. Touch alters “the dimensions of the body, inciting it to move in excess of its-self toward the world” (Manning, 2007, p. xiii.) To develop and elaborate on her idea of touch in the human realm, Manning uses her own striking example, that of the tango—a dance which emerges out of and expresses displacement, loss, and desire all at once. The tango is about the pain of disconnection and the desire for communication, personally, and culturally and politically.^4^ It involves a state of becoming through alterity since it resides neither entirely on its own terrain nor on the terrain of the other. Much that Manning describes in her analysis of the tango—this most physically close and sensual of dances—found echoes in our group intra-actions. The affective energies and repetitive yet changing moves of the tango are similar to those animating and fuelling our daily get-togethers.

To sum up, the process philosophers we think-with—Barad, Massumi and Manning—trouble the common-sense idea of touching. They stress the relationality, responsiveness, and indeterminacy of touch. For them, as for us in this article, virtual touch, although encompassing the digital, is, above all, about potential.

### Montage: Touch Fragments

In this section, we present a montage of matterings, of fact, concern, and of care (Haraway, 2016; Juelskjær & Schwennesen, 2012; Puig de la Bellacasa, 2017)—touch fragments of our daily readingwritings^5^ in response to Barad’s text, the specifics of each of our experiences of coronatime. Drawing on Walter Benjamin’s materialist methodology, Barad (2017) suggests that the process of montage can be compared with picking up crystal fragments and examining them through different light rays. This allows insights to “flash up” in new constellations. Barad (2017) notes that writing as montage enables shifting diffraction patterns where insights can be read “through one another, allowing the reader to explore various crystalline structures that solidify, if only momentarily” (p. 22). We selected fragments of each of our free writings which we wrote daily after reading Barad’s texts, as well as a collaborative artwork in a series of diffractive maneuvers that provoked past/present/future thoughts and experiences. The writing fragments in our montage are not linear or analogous, but are intended to allow the reader “to discover in the analysis of the small individual moment the crystal of the total event” (Benjamin cited in Barad, 2017, p. 30).

Denise, Saxonwold, Johannesburg: What fascinates me is the surprising affordance of Zoom for enabling intimacy during coronatime. The Speaker View has an affective intensity, like a cinematic close-up, bringing the speaker into my space, very close to me, less than half a meter away. It transforms the speaker into a *person.* Not having seen Viv for a long time, I now see her looking straight at me, as I look at her. I see her blonde ringlets shining in the shaft of sunlight, sometimes wet after her daily sea swim. I see her thinking eyes look upward as her lips mouth words and ideas. I hear her voice undisturbed by ambient sound. I feel close to her, as if I can touch her, closer than if I was with her. If we, for example, sat at a table at Bootleggers coffee shop, across from each other, we would not be this close; if we walked along the Sea Point promenade, watching the wild waves crashing and discussing the diffraction patterns, we would be alongside, not having a frontal view. I feel touched by her, and perhaps she feels touched by me too. We spend more time together here than on my brief visits to Cape Town. There is more time now than pre-COVID; we meet every day rather than once a year. And, in Gallery View, I am part of a group of seven women, from different South African cities and from Brugge. I enjoy their different looks, voices, habits, skills, idiosyncrasies, and ways of thinking and relating. I am locked down in my home and privileged to have a home. I barely go out, but I am comforted, provoked, and inspired by the company and theatrics of our daily get-togethers. This digitized experience, new for me, is full of wonder, as well as of wanderings!

Alex, Woodstock, Cape Town: Our lockdown spacetime matterings are constituted through our entangled readings, writings, and thoughts. Viv is my PhD supervisor and has keen interests in notions of slow-learning so we read Barad’s texts slowly to one another, taking turns. I find the concepts complex as we delve into the fine details while doing our own Gedanken^6^ experiments to make sense of the theory, and worldly COVID matterings.

Over time, our encounters have become more personal, as we’ve been touched in numerous ways. Through the repetitive, daily laughing and chatting and figuring out Barad, our supervisor/student relations have shifted into more intimate spaces. I feel a sense of holding each other as we journey through the different stages of lockdown while trying to make sense of the virus, cooped up at home with curfews and strict social distancing rules. In a Baradian sense, it feels like the virus is the stranger-within, affecting our usual ways of being, of reading and getting together, as well as our intra-actions with our broader communities. Like the Schrödinger cat^7^ experiment, the deadly virus is an indeterminate phenomenon.

The indeterminacy of this time has extended our intra-actions to that of caring for one another’s well-being. It has made me think more deeply about caring for myself and my safety as a diabetic with corona. I feel my precarity in terms of the risky connection between COVID and diabetes, as well as facing difficult decisions regarding my son’s safety such as whether he should be attending school or not. My concerns extend to the safety of my students.

Veronica, Rondebosch, Cape Town: Denise’s story about her Jewish Holocaust background, and how her mother would not say one word about it, reminded me of my own family’s silences. Denise confessed that she did not normally disclose this topic, buried deep inside her. I was touched by these hauntings. The violences of past/present/future bring insights into current agonizing circumstances.

My interest and work in women’s health with medical undergraduate students foreground professional response-abilities. Alongside the COVID pandemic, South Africa acknowledges a second prevailing pandemic that has intensified in terms of increased acts of gender-based violences (Ellis, 2020). Public health restrictions to stay at home conflict with the precariousness of home safety for many. Can gender-based violence be considered as a void in medical training? Is there a repulsive force that silences these undesirable human violences? Reading Barad’s texts together with the group heightened my understanding of the multiple perspectives of a-void-ance.

COVID has hit me with two hard blows. Although I had bought two pulse oximeters^8^ for protection against possible severe COVID symptoms, these finger-touch machines could not intervene in two related personal events. First, hearing from my doctor daughter that she tested COVID-positive in Johannesburg, a city far away from my home. Second, the death of a dog that was in our care while her owner was trapped by COVID regulations in the United Kingdom. The distancing and isolation at the brink of living/dying escalated to a harsh reality for me, resonating with those thousands of people worldwide suffering COVID consequences.

Lieve, Brugge, Belgium: Trying to stay in touch with South Africa, COVID-19 and technology created the illusion it did not matter that my Zoom window was located in Belgium. Living at a distance and social distancing are overcome by online meetings, as we keep reaching out to one another. “We have to meet the universe halfway, to move toward what may come to be in ways that are accountable for our part in the world’s differential becoming. All real living is meeting. And each meeting matters” (Barad, 2007, p. 353). Our different stories were not only bringing us closer, they were also cutting us apart.^9^ I was touched by being up in the North, the cradle of world wars, colonization, and travelers spreading the virus as capitalist neoliberals. Taking turns in embodying the stranger within, accepting the invitation to dance, and being fearful of catching the same virus intensified our encounters. Meeting and becoming with the group certainly affected me and was the start of différance.^10^ I did not anticipate that I would find renewed interest in my own history to better understand what the South was holding the North accountable for, and discovering local history which I had not noticed before. I think this was the most important difference from other reading groups which tend to be more about processing texts. In this group, it is more about becoming-with each other through affecting and being affected and developing relationships that matter.

Viv, Kenilworth, Cape Town: This reading group is a comfort in its daily reiterative meetings—something to look forward to. There is an ethos in the group which allows us to touch and be touched by each other—entangled with the machine and the internet which usually makes it possible to connect with each other, except when there are power failures.

My well-worn, dog-eared, and puppy-bitten paperback book is testament to the many times I have read and re-read Barad. However, our reading group has significantly increased the depth of my understanding of her work, as it is through reading the *whole* text aloud, and being read to, that I gain a better grasp of difficult concepts.

My identity has been undone and keeps being reconstituted through reading Barad and through our intra-actions. My previous understandings of the world are unmoored through our collaborative reading and sensemaking of Barad, dislodging notions of human intentionality, separability, the internal and external and representationalism. Also, concepts I had eschewed as positivist—phenomena, objectivity, measurement, and causality—are reconfigured by Barad. I now consider them from different angles. The void, virtuality, and in/determinacy shake the very foundations of my prior existence—that “there is no determinate fact of the matter” is what unravels prior complacency the most. This is both unsettling and exhilarating. Corona is also undoing us as it is reinforcing the precarity and indeterminacy of life on earth and making us aware of the importance of both living and dying well in the world. I am reminded how those who are noncontemporaneous are part of us when we touch each other. How is Denise’s past part of the stranger within that we touch? How does this affect Lieve who lives near Germany and how is it different for us located in South Africa? What about Belgian and British and other forms of colonialism in which we are implicated? If every being is already threaded through with an infinite alterity, is there anything that is not touchable? The ghosts of our past, the differences between the haves and the have nots, and the damaged environment which have arisen from past actions, have all given rise to the coronavirus.

Nike, Tamboerskloof, Cape Town: In the era of strict COVID lockdown and self-isolation, the Corona Reading Group members are paradoxically more intimately connected than ever before: “We” transgress the boundaries of bodies (both human and other) as our voices enter one another’s heads through earphones. The rhythm of our fingertips striking keyboards resonates in each other’s homes across the world. We listen to each other reading Barad’s words, as our eyes re/turn to the text again and again. In this virtual room, Euclidean scale and proportion have little bearing as we see each other through seven portals connecting us. Unlike in real time and space where we are mindful not to transgress “personal space,” our coming together online has different sensibilities. For example, touching means something else here: if I wanted to, I could tickle your faces and you wouldn’t feel it. We touch each other differently here. Our words touch each other, as do our voices, facial expressions, and our silences.

I have been taking regular walks on the promenade . . . usually after we have met as a group. Our conversations, our questions, our readings segue into these walks . . . I watch the waves moving, swelling, receding, bubbling up, and within them glimpse traces of our discussions that I so wish we could continue. It is frustrating and I try and reach out to you in Johannesburg and Belgium with photos and video-clips in an attempt to prolong and extend our comings together.

Kath, Greenstone Hill, Johannesburg: “What is the measure of closeness?” (Barad, 2014, p. 153). We touch one another with our words, smiles, laughs, silences, and thoughts. Our reading group conversations often flow into the WhatsApp group, with never-ending sharing of comments, thoughts, ideas, and images at all times of the day and night, extending and expanding connections from one machine to another and one time and place to another, one intra-action to the next, inextricably entangled.

When I joined the reading group, I had only known one of my six other Zoommates. But with time and our virtual connection, I came to know everyone. We celebrated our birthdays with virtual online parties. These strengthened our bonds as we got to know each other a little better and admired Lieve’s famous birthday tables. How did we go from reading Barad to celebrating birthdays when not all of us had met in the “flesh”? What does this say about machinic agency and connections, about touch and virtual touching?

The Corona Reading Group is like a “safe space,” where we get to talk to one another about our challenges, anxieties, hopes, and fears, about this special time that we are living through—the coronatime—while simultaneously engaging with academia differently.

Barad (2014, p. 155) says, “Touch, for a physicist, is but an electromagnetic interaction.” For me, touch, in the Corona Reading Group, is but a “matter of response. Each of ‘us’ is constituted in response-ability. Each of ‘us’ is constituted as responsible for the other, as being in touch with the other” (Barad, 2014, p. 161).

### Of cables and webs, collective photomontage, with acknowledgment to *Maman* of Louise Bourgeois

Prompt #10 of the Massive_Micro project (Markham et al., 2020) invited us to think about machines as agentic, technology as relational and automation as mediators, to better understand the connections, juxtapositions, and relations of machines and humans during the time of the COVID pandemic.^11^ We decided to respond collaboratively to the prompt because it offered an opportunity for us to move beyond our screens and reach toward each other’s spaces through the connections and affordances that the various technologies offer us.

**Figure 1.**
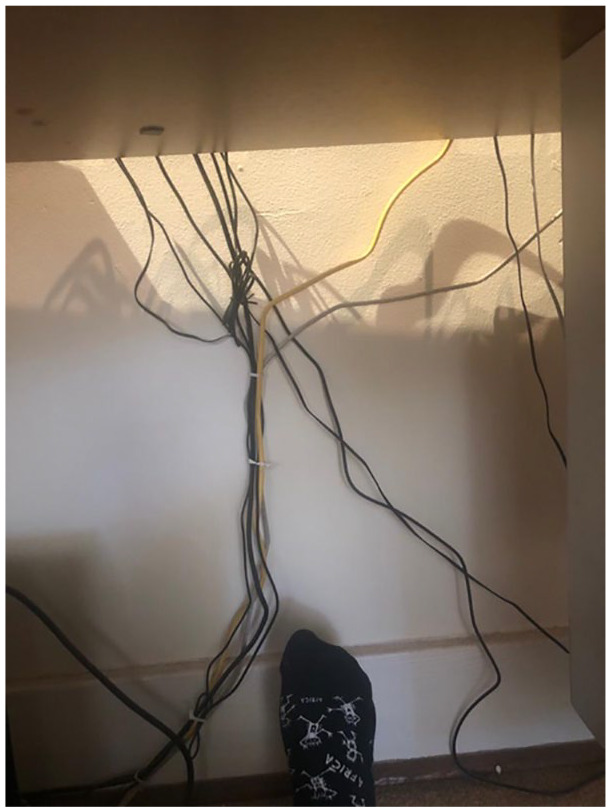
Cable underneath Denise’s desk. *Note.* Photo: Denise Newfield.

**Figure 2.**
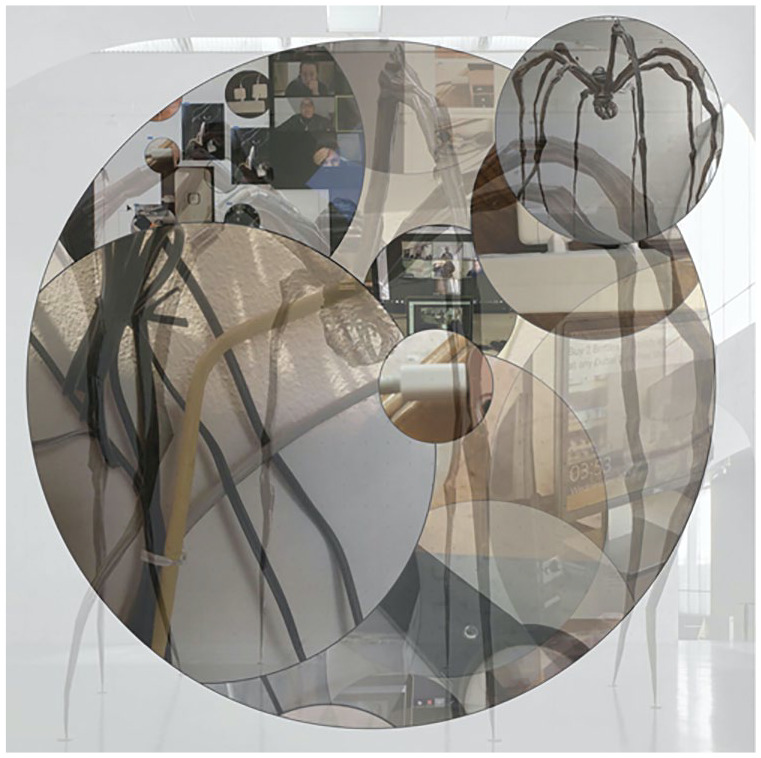
Of cables and webs. *Note.* Screenshot: Nike Romano.

To begin, we photographed our machines and their appendages—the cables, the chargers, the attachments, the speakers, and the headphones—that provide the means through which we connect. Sharing our images in a shared WhatsApp group, we entered our respective homes. The Zoom recording of this session reveals an exuberant and animated encounter as we shifted from the usual talking heads in the machine format, to a virtual playdate in which we marveled at children’s toys, a grandfather’s portmanteau, dusty floorboards, and a map of Africa, to mention a few. Curiously, there was one image of the computer cables under Denise’s desk (see Figure 1) that bore an uncanny resonance with Louise Bourgeois’ sculpture, *Maman* (see Figure 2). As will be elaborated below, this image was to play a pivotal role in the creation of our shared response.

Using Zoom’s shared screen option and working in InDesign, our initial plan was to place images alongside one another in a series of squares that, much like a cottage window, would frame the various views. However, the rectilinear grid, that embodies the x and y binary logic of modernist thinking, felt like an imposing structure that would restrict our flow and commitment to the immanence of the specific material-discursive encounter (Krauss, 1979). Rather than setting up a Show & Tell scenario, we turned toward a more performative arrangement that enacted rather than represented our ongoing entangling web.

In theorizing the network as a mode of being, that he calls the Arachnean, Deligny thinks with the spider and the weaving of its web. This analogy signals two important concerns that have bearing on our group. The first concerns the precarity of networks that, like the spider’s web, are constantly at risk of disintegrating. The second highlights how to thrive, like the spider’s web that needs something to attach to, in addition to staying intact, the network needs to attach, to touch, to hold onto something (Deligny, 2015, p. 21).

Deligny also emphasizes how the process of weaving the web matters more to the spider than catching the fly. He writes,

What is Arachnean about the network, it being understood that the Arachnean is not a having but an incessant finding, a discovery punctuated by surprises, these surprises being very peculiar coincidences that can only occur if wanting remains confined to what it can do and what is of concern to it (Deligny, 2015, p. 75).

Throwing caution to the wind, we eschewed a predetermined plan and turned toward an indeterminate process that allowed the entanglement to weave itself (Deligny, 2015). Our working together through a shared screen evoked a tentacular arrangement like that of the spider with its many eyes and limbs.

Instead of juxtaposing rectangular images alongside each other, we opted to contain them in circles as these were more evocative of the themes, such as planets, bubbles, viruses, and voids, that mattered. We also adjusted opacity levels which enabled a layering effect that revealed images through each other. In the process of touching each other, the photographs metamorphosed from discrete objects to becoming-with each other that materialized the montage entitled *Of cables and webs*, with acknowledgment to *Maman* of Louise Bourgeois.

Like the images, our touching draws us toward constant becoming through movement. Understood in this way, rather than seeing the self as discrete subjectivities that we return to, we become “a proliferation of vectors of intensity that emerge through contact” (Manning, 2007, pp. 136–137).

### Affect, Response-Ability, and Justice-to-Come

This article has argued that the period of forced isolation during the disruptive and uncertain months of coronatime materialized the unexpected affective potential of virtual touching. Our initial experience of social distancing and the silence of coronatime seemed to void life. It is in this non/presence of life as we knew it that we felt compelled to meet each other halfway in the void of lockdown where we read Barad à haute voix (Deleuze, 1980) and touched one another in online spaces.

We swerved off familiar pedagogical paths, wandered, open to the unknown, and then returned and re-turned, “not as consecutive moves but as experiments in in/determinacy” (Barad, 2014, p. 155). The process differed from traditional structured or preconstituted methods for pedagogical practices and from representational research. The dynamics of the process were energized as much by questioning and dissensus as by robust creative engagement. Given that the PhD process is known to be a lonely journey, the value of working and learning together/apart, with care and generosity, touched on new possibilities for supervisors and students. As we moved toward the other, being open to the more-than, our virtual touching expanded our capacities to affect and be affected. We are not proposing that our particular experience can be generalized, but rather show how the potentialities of this reading group arose out of a particular configuration, a specific entanglement.

For us, touching manifests through quantum physics/Baradian and Deleuze & Guattari/Massumi/Manning notions of touch—as yearning, as desire, as reaching toward, as affecting and being affected, thereby determining what our bodies can be and do. In so doing, virtual touch engendered a crossing of ontological thresholds through affective entanglements between ourselves and our specific contexts, Barad’s texts and COVID-19.

During these iterative encounters, we found that virtual touching revealed matter’s agential capacities for imaginative, desiring, and affectively charged forms of bodily engagements. Lively play and desires engendered infinite sets of possible intra-actions (Barad, 2015, p. 158) in the “frothy virtual soup of indeterminacy” (Barad, 2019, p. 530). Touching produced different ways of being and becoming-with human and nonhuman others.

Our touching became a dance across cities and countries, an invitation and a response, like Barad’s description of the yearning of lightning by stepped leaders which reach toward filaments from the earth—an enlivening adventure fueled by desire and response-ability. We moved together as a diffractive multiplicity emerging out of affinities, attractions and magnetisms as well as uncertainties, divergences, fissures and dissensus. The choreography was indeterminate—not planned in advance—the tempos, rhythms, and flows finely attuned, they moved through each other, we moved through each other, undoing dichotomies of self and other, finding opportunities for respite, retouch, and reparation.

The animated process of “cutting and pasting” our photomontage, *Of Cables and Webs*, drew us together and in so doing redrew us as “a proliferation of vectors of intensities” (Manning, 2018, p. 136). As strangers within, the backlit images shone through each other, troubling the linearity of time and fixity of space, illuminating the more-than of their expansive diffractive intra-action. Similarly, our encounters with theory have thickened; Barad (2015) notes how theory is about being in touch—an openness to the murmurings of the world. Our collective reading of mind-blowing concepts and our current experiences of coronatime undo our identities—ourselves—which as Barad points out are never “our” or “selves,” as there are no boundaried units. “We” are undone and redone through touching Barad, each other, corona.

Touching has opened up innovative ways for doing academia differently. Our care-full intra-actions activate different forms of touching between self and other that are unexpectedly invigorating, inspiring, and rewarding. They create expansive understandings of each other, ourselves, different contexts, and theoretical concepts in texts that we touch upon and are touched by, marking our bodies.

Our responsive caring is a reciprocal move toward the political and hauntological response-abilities of social and planetary justice-to-come:

In an important sense, in a breathtakingly intimate sense, touching, sensing, is what matter does, or rather, what matter is: matter is condensations of response-ability. Touching is a matter of response. Each of “us” is constituted in response-ability. Each of “us” is constituted as responsible for the other, as being in touch with the other (Barad, 2014, p. 161).

Although touch is difficult to grasp (literally and figuratively), it is what matter is, does, and undoes and it is what matters during coronatime. All touching matters, and all matters touch.

## Notes

1. Intra-action is different from interaction. Barad (2007) uses the neologism “intra-action” to describe how everything is predicated upon a relational ontology where entities or individuals come into being through relationships. With interaction, preexisting entities or individuals are drawn into relationships with one another.

2. Barad’s use of slashes between words troubles dichotomies and binary distinctions. In this instance the slash indicates that they are neither together *and* apart, nor together *or* apart, but rather in a state of indeterminacy.

3. Diffraction refers to what happens when any kind of waves—water, light or sound—bend and spread out when they hit a barrier, or combine when they overlap.

4. Tango, unlike its present form as a ballroom dance, originates as an improvised “dance of exiles” of the descendants of African slaves, and Italian and Spanish immigrants to Buenos Aires (Savigliano cited in Shafie, 2019, p. 36).

5. Sarah Truman (2016) and Vivienne Bozalek (2017) question the separation of reading and writing in their work and refer to readerlywriters and writerlyreaders in an attempt to bypass the binaries between reading and writing.

6. Gedanken experiments are hypothetical thought experiments rather than actual laboratory experiments which consider theory for thinking through an experiment’s consequences.

7. Schrödinger’s cat was a thought experiment devised by quantum physicist Erwin Schrödinger to understand the difficulties of the nature of measurement. A hypothetical cat is placed in a sealed box with a flask of poison and a radioactive source. The cat is in an entangled state of indeterminacy regarding being alive or dead. There is “no determinate fact of the matter about its condition” (Barad, 2014, p. 251).

8. A pulse oximeter is an electronic device that connects with an individual’s finger to measure oxygen saturation levels in the blood—an important indicator of the severity of respiratory failure associated with COVID-19.

9. Cutting together apart (one move) troubles the notion of dichotomy—cutting into two—and assumes a state of indeterminacy, where two states coexist at the same time.

10. Différance, a neologism of Derrida’s means both same and other, and according to Kirby (2018, p. 125), “becomes an ‘always already not yet’ that mangles the coordinates that difference and causality assume.”

11. Measuring (927 × 891 × 1,024 cm), *Maman* is one of the world’s largest sculptures and fits well with the Massive-Micro theme. The spider is a homage to the artist’s mother who worked in the tapestry industry. It symbolizes her mother’s strength and the protective role that she played in Bourgeois’ life.

## References

[bibr1-1077800420960167] Ahmed S. Stacey J. (Eds.). (2003). Thinking through the skin. Routledge.

[bibr2-1077800420960167] Barad K. (2007). Meeting the Universe Halfway: Quantum physics and the entanglement of matter and meaning. Duke University Press.

[bibr3-1077800420960167] Barad K. (2014). On touching—The inhuman that therefore I am (v1.1). In Witzgall S. Stakemeier K. (Eds.), Power of material/politics of materiality (pp. 153–164). Diaphanes.

[bibr4-1077800420960167] Barad K. (2015). TransMaterialities: Trans*/matter/realities and queer political imaginings. GLQ: A Journal of Lesbian and Gay Studies, 21(2–3), 387–422.

[bibr5-1077800420960167] Barad K. (2017). What flashes up: Theological-political-scientific fragments. In Keller C. Rubenstein M. (Eds.), Entangled worlds: Religion, science and new materialisms (pp. 21–71). Fordham University Press.

[bibr6-1077800420960167] Barad K. (2019). After the end of the world: Entangled nuclear colonialisms, matters of force, and the material force of justice. Theory & Event, 22(3), 524–550.

[bibr7-1077800420960167] Bozalek V. (2017). Slow scholarship in writing retreats: A diffractive methodology for response-able pedagogies. South African Journal of Higher Education, 31(2), 40–57.

[bibr8-1077800420960167] Deleuze G. (1980). Spinoza: The velocities of thought [Seminar at the University of Paris, Vincennes-St. Denis, 1980-1981]. https://deleuze.cla.purdue.edu/seminars/spinoza-velocities-thought/lecture-02

[bibr9-1077800420960167] Deligny F. (2015). The Arachnean and other texts [Burk D. S. Porter C., Trans.]. Univocal Publishing.

[bibr10-1077800420960167] Derrida J. (1994). The Specters of Marx: The state of the debt, the work of mourning, and the new international (Kamuf P., Trans). Routledge.

[bibr11-1077800420960167] Ellis E. (2020, June, 16). Gender-based violence is South Africa’s second pandemic, says Ramaphosa. Daily Maverick. https://www.dailymaverick.co.za/article/2020-06-18-gender-based-violence-is-south-africas-second-pandemic-says-ramaphosa/#gsc.tab=0

[bibr12-1077800420960167] Genosko G. (2009). Felix Guattari: A critical introduction. Pluto Press.

[bibr13-1077800420960167] Haraway D. (2016). Staying with the trouble: Making kin in the Chthulucene. Duke University Press.

[bibr14-1077800420960167] Jewitt C. Price S. Mackley K. Yiannoutsou N. Atkinson D. (2020). Interdisciplinary insights for digital touch communication [eBook]. 10.1007/978-3-030-24564-1

[bibr15-1077800420960167] Juelskjær M. Schwennesen N. (2012). Intra-active entanglements: An interview with Karen Barad. Kvinder, Køn & Forskning, 1–2, 10–23. 10.7146/kkf.v0i1-2.28068

[bibr16-1077800420960167] Kirby V. (2018). Un/Limited Ecologies. In Fritsch M. Lynes P. Wood D. (Eds.), Eco-deconstruction: Derrida and environmental philosophy (pp. 121–140). Fordham University Press.

[bibr17-1077800420960167] Krauss R. (1979). Grids– Rosalind Krauss (Vol. 9, pp. 50–64). https://creativitycontext.wordpress.com/key-texts/grids-rosalind-krauss/

[bibr18-1077800420960167] Manning E. (2007). Politics of touch: Sense, movement, sovereignty. University of Minnesota Press.

[bibr19-1077800420960167] Manning E. (2018). Politics of touch. Conference Hold Me Now—Feel and Touch in an Unreal World, Stedelijk Museum, Amsterdam. https://www.youtube.com/watch?v=l7yQaicWD_M

[bibr20-1077800420960167] Markham A. Harris A. (2020). Massive and microscopic sense-making in the time of COVID. Call for Expressions of Interest. https://futuremaking.space/call-for-participation/

[bibr21-1077800420960167] Markham A. Harris A. Luka M.E. (2020). Massive and microscopic sensemaking in times of COVID-19. Qualitative Inquiry.

[bibr22-1077800420960167] Marks L. (2002). Touch: Sensuous theory and multisensory data. University of Minnesota Press.

[bibr23-1077800420960167] Massumi B. (2011). Semblance and Event: Activist philosophy and the occurrent arts. The MIT Press.

[bibr24-1077800420960167] Massumi B. (2015). Politics of affect. Polity Press.

[bibr25-1077800420960167] Puig de la Bellacasa M. (2017). Matters of care: Speculative ethics in more than human worlds. University of Minnesota Press.

[bibr26-1077800420960167] Shafie R. (2019). Social Tango dancing in the age of neoliberal competition [Unpublished doctoral dissertation]. University of California.

[bibr27-1077800420960167] Sundén J. (2014). Steampunk practices: Time, tactility, and a racial politics of touch. Ada: A Journal of Gender, New Media, and Technology, 5. http://adanewmedia.org/2014/07/issue5-sunden/

[bibr28-1077800420960167] Truman S. (2016). Intratextual entanglements: Emergent pedagogies and the productive potential of texts. In Snaza N. Sonu D. Truman S. E. Zaliwska Z. (Eds.), Pedagogical matters: New materialisms and curriculum studies (pp. 91–108). Peter Lang.

[bibr29-1077800420960167] Young E. B. Genosko G. Watson J. (2013). The Deleuze and Guattari dictionary. Bloomsbury.

